# Alantolactone: A Natural Plant Extract as a Potential Therapeutic Agent for Cancer

**DOI:** 10.3389/fphar.2021.781033

**Published:** 2021-11-26

**Authors:** Yuan Cai, Kewa Gao, Bi Peng, Zhijie Xu, Jinwu Peng, Juanni Li, Xi Chen, Shuangshuang Zeng, Kuan Hu, Yuanliang Yan

**Affiliations:** ^1^ Department of Pathology, Xiangya Hospital, Central South University, Changsha, China; ^2^ National Clinical Research Center for Geriatric Disorders, Xiangya Hospital, Central South University, Changsha, China; ^3^ Department of Pathology, Xiangya Changde Hospital, Changde, China; ^4^ Department of Pharmacy, Xiangya Hospital, Central South University, Changsha, China; ^5^ Department of Hepatobiliary Surgery, Xiangya Hospital, Central South University, Changsha, China

**Keywords:** Alantolactone, anticancer effects, cancer, signaling pathways, regulatory factors

## Abstract

Alantolactone (ALT) is a natural compound extracted from Chinese traditional medicine *Inula helenium L.* with therapeutic potential in the treatment of various diseases. Recently, *in vitro* and *in vivo* studies have indicated cytotoxic effects of ALT on various cancers, including liver cancer, colorectal cancer, breast cancer, etc. The inhibitory effects of ALT depend on several cancer-associated signaling pathways and abnormal regulatory factors in cancer cells. Moreover, emerging studies have reported several promising strategies to enhance the oral bioavailability of ALT, such as combining ALT with other herbs and using ALT-entrapped nanostructured carriers. In this review, studies on the anti-tumor roles of ALT are mainly summarized, and the underlying molecular mechanisms of ALT exerting anticancer effects on cells investigated in animal-based studies are also discussed.

## 1 Introduction

Cancer is characterized by a very high incidence rate and fatality rate, and seriously affects human health (Fidler et al., 2017). Cancer maintains the malignancy by affecting the development of the embryo and destroying the repair mechanisms (Guan et al., 2020). It has been found that genomics-based assays can be used in clinical therapy, such as targeted treatment and antitumor vaccines (Berger and Mardis, 2018). Currently, surgical resection, radiotherapy, and chemotherapy are the main effective modalities for curing cancers. Chemotherapy uses anti-cancer compounds and medicine to attenuate cancer development (Seo et al., 2009). However, treatment failure and side effects are common in chemotherapy. Therefore, new drugs with better therapeutic effects and fewer adverse effects are needed for cancer treatment.

Nowadays, alantolactone (ALT), a natural herb compound derived from the traditional Chinese medicinal *Inula helenium L.*, has attracted extensive research attention because of the therapeutic potential in cancer treatment (Mi et al., 2014). It has been revealed that ALT can exhibit anti-inflammatory and anti-tumor activities through modulating the abnormal signaling pathways in cancer cells (Gierlikowska et al., 2020; Babaei et al., 2021). For example, mitogen-activated protein kinases (p38 MAPK) and NF-κB signaling pathways are significantly attenuated by ALT, inhibiting cell viability and promoting cell apoptosis in lung cancer cell lines NCI-H1299 and Anip973 (Liu et al., 2019). And a recent study firstly reported that ALT could suppress the activation of YAP1/TAZ, leading to the inhibition of cancer cell growth (Nakatani et al., 2021). ALT could downregulate the serine/threonine kinase Aurora-A through directly binding to the interface pocket of Aurora-A-TPX2 complex, weakening several cancer-associated biological behaviors, including centrosome amplification, chromosomal instability and oncogenic transformations (Bhardwaj and Purohit, 2020; Nadda et al., 2020). Furthermore, with no obvious side effects, ALT could synergistically enhance the cytotoxic effects with other anti-cancer agents, such as oxaliplatin (Cao et al., 2019) and olaparib (Wang et al., 2020) *in vivo* and *in vitro*.

**FIGURE 1:**
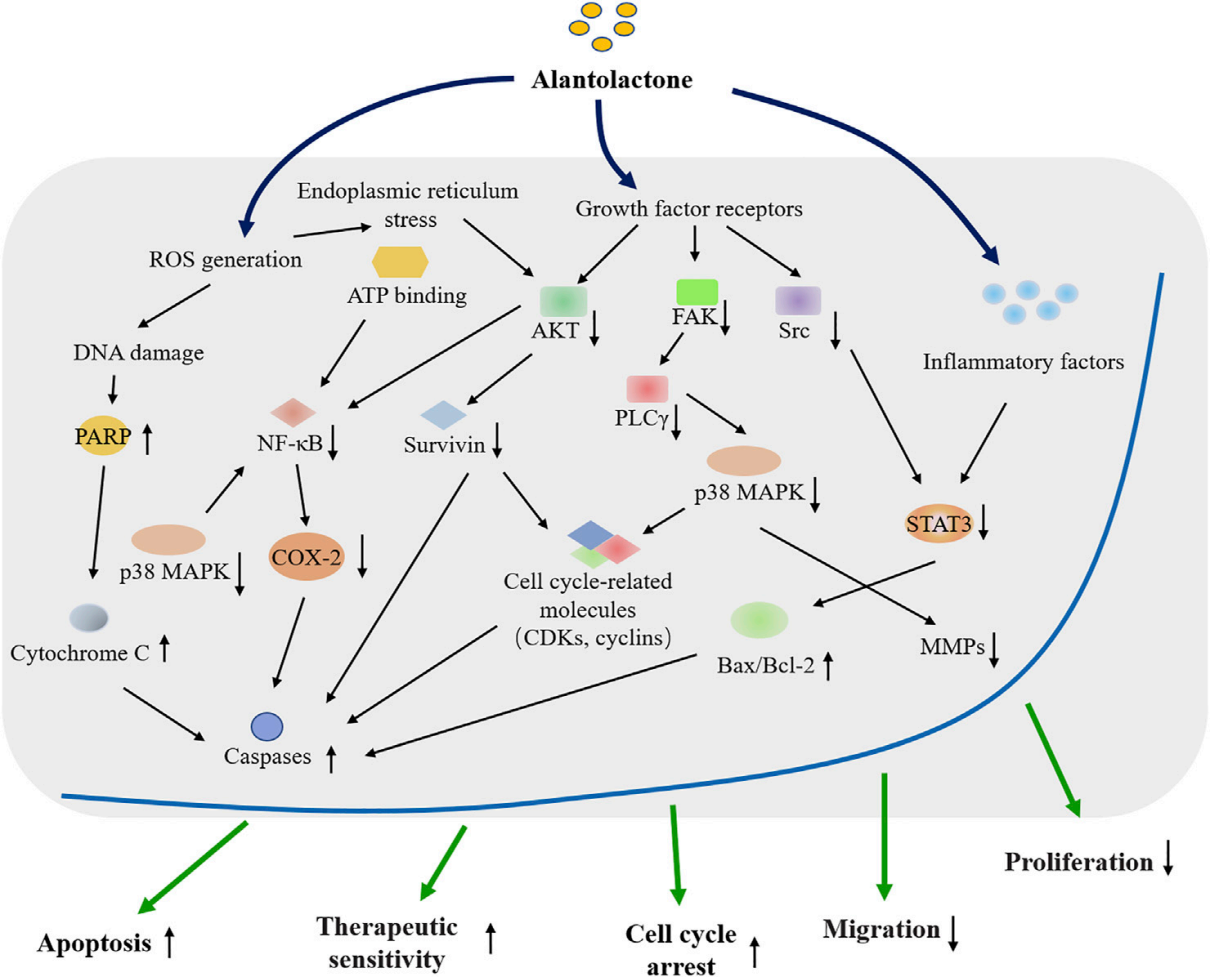
Overview of the cytotoxic effects of the natural compound Alantolactone on cancer research and therapy.

In this paper, the findings regarding the antagonistic effects of ALT in various cancers are summarized, and the underlying mechanism of ALT anticancer activity is explored (Figure 1, Tables 1, 2). Besides, to explore the practical values of ALT in future clinical applications, the safety and efficacy of ALT are also discussed.

**TABLE 1:** The anticancer activities and the underlying mechanisms of alantolactone *in vitro*.

Cancers	Cell lines	Modulated factors	Biological effects	References
Liver cancer	HepG2 cells	Bcl-2, caspase-3, STAT3	Inducing apoptosis, inhibiting cell proliferation, inducing G2/M phase arrest	Khan et al. (2013)
		Bcl-2, NF-κB, p53, Bax, caspase-3/8/9, t-Bid		Lei et al. (2012)
		p21, cyclin A1 cyclin B1, caspase-3, PARP		Kang et al. (2019)
Colorectal cancer	SW480 and SW1116 cells, non-cancer BEAS-2B and L-O2 cells	Bcl-2, Bax, caspase-3, p21	Inducing G1 cell cycle arrest, inducing apoptosis, inhibiting cell proliferation	Ding et al. (2016)
	Murine CT26-FL3 cells, Murine breast cancer 4T1 cells	Bcl-2, Bcl-xL		Zhang et al. (2019a)
	HCT116 and RKO cells	JNK, p38, MAPK, Ki-67		Cao et al. (2019)
	HCT-8, L02, HEK 293 T cells	Cripto-1, ActRIIA, activin, SMAD3, p21		Shi et al. (2011)
	RKO cells	MMP, Bcl-2, Bax, caspase-3/9, cytochrome c		Zhang et al. (2013)
Breast cancer	McF-7 cells	Bcl-2, Bcl-2-associated X protein, p53, p65, caspase-3, caspase-12, MMP-2, MMP-7, MMP-9, p38, MAPK, NF-κB, Nrf2	Inhibiting cell proliferation, inducing apoptosis, inhibiting motility, migration and tube formation, causing cell cycle arrest	Liu et al. (2018a)
	HUVECs, MDA-MB-231 cells	VEGFR2phosphorylation, PLCγ1, FAK, Src, Akt		Liu et al. (2018b)
	Triple-negative breast cancer (TNBC) cells	Bcl-2, Bax, caspase-3, CyclinB1, Cdc2, ATF4, CHOP, ki-67		Yin et al. (2019)
	MDA-MB-231, MCF-7 cells	Bax/Bcl-2, MMP, cytochrome c, caspase 9/3, PARP, MAPKs, p-NF-κB, p65, p-STAT3, NF-κB, AP-1, STAT3		Cui et al. (2018)
		STAT3, MAPKs, NF-κB, IL-6, EGFR, cyclin D1, c-Rel, p65, p50, JNK/AP-1		Chun et al. (2015)
Lung cancer	NCI-H1299 and Anip973 cells	Bcl-2, MMP-9, MMP-7, and MMP-2, β-actin, p38MAPK, NF-κB	Inducing cell apoptosis, suppressing migration, invasion, and colony formation, inhibiting cell proliferation	Liu et al. (2019)
	SK-MES-1 cells	Caspases-8, -9, -3, PARP, Bcl-2, Bax, CDK4, CDK6, cyclin D3, cyclin D1, p21, p27		Zhao et al. (2015)
	A549 cells and NcI-H520 cells	Xiap, survivin, caspase-9, caspase-3, PARP, ATF4, eIF2α, CHOP, Bcl-2, Bax, STAT3, iNOS, COX-2, MMP-9		Maryam et al. (2017)
		PI3K/Akt, ER, p21, cyclin A2		Wang et al. (2019a)
Leukemia	HL-60 cells	Cytochrome c, Bax, caspase-3, PARP	Inducing apoptosis, inhibiting cell proliferation, inducing cell cycle arrest	Pal et al. (2010)
	THP-1 cells	STAT-3, survivin, Bcl-2, Bcl-xL, Bax, cl-caspase-3, cl-PARP, cytochrome c		Ahmad et al. (2021)
	K562 and K562r cells	NF-κB, p65 Bcr/Abl protein, caspase-3, PARP-1		Wei et al. (2013)
	CML blast cells			
	BV173 and NALM6 cells	AP2M1, Beclin1, LC3-II/LC3-1, p62, Bax, cleaved caspase 3, cytochrome C, Bcl-2		Shi et al. (2020)
	B-ALL cell lines	PARP-1, capase-3, caspase-8, caspase-9, NF-κB, BCR-ABL, EGFR		Xu et al. (2019b)
Pancreatic cancer	MIA PaCa-2 and PANC-1 cells	TFEB, CTSB/CTSD	Inducing apoptosis, improving chemosensitivity, inhibiting proliferation, inhibiting migration	He et al. (2018)
	BxPC-3, AsPC-1, and PANC-1 cell lines	STAT3		Zheng et al. (2019)
	PANC-1 and SW1990 cells	Caspase 3/7, Bak, Bcl-2, Mcl-1, XIAP, STAT3		Yan et al. (2020)
Gastric cancer	SGC-7901 and BGC-823 cells	TrxR1, p38MAPK, p38, Ki-67, Bcl-2	Inhibiting proliferation, inducing apoptosis	He et al. (2019a)
		Bcl-2, Bax, cleaved PARP, cyclin D1, p21, p27, AKT, cyclin-dependent kinase inhibitor 1, cyclin-dependent kinase inhibitor 1B		Zhang and Zhang (2019)
		Bax, Bcl-2, p53, MMP-2, MMP-7, MMP-9, NF-κB, p38MAPK, p65		He et al. (2019b)
Cervical cancer	HeLa cells	Bcl-2, Bax	Inhibiting proliferation, inducing apoptosis	Jiang et al. (2016)
		Caspase-3, Bax, Bcl-2, NF-κB		Zhang et al. (2019b)
		TrxR, caspase 3		Zhang et al. (2019a)
Glioblastoma	U87 and U251 cells	IKKβ/NF-κB, p50, p65, p300, COX-2, cytochrome c, cyclin D1, CDK4, MMP-2, MMP-9, caspase-3/9, PARP, Bax, Bcl-2	Inhibiting cell growth, inducing apoptosis	Khan et al. (2012), Wang et al. (2017)
Osteosarcoma	U2OS and HOS cells	PI3K/AKT, cyclin D1, p27, Bcl-2, Bax, cleaved caspase-3/8, MMP-2, MMP-9	Inhibiting proliferation, promoting apoptosis	Zhang et al. (2019c)
Multiple myeloma	RPMI8226, NCI-H929, IM9, MM1R, MM1S, OPM2 and U266 cells	ERK1/2, IL-6, VEGF, caspase-3/8/9, Bcl-2, Bax, survivin, cyclin D, cyclin E, CDK 2, CDK 4, MAPK	Inhibiting proliferation, inducing G1 phase arrest, inducing apoptosis	Yao et al. (2015)

**TABLE 2:** The anticancer activities and the underlying mechanisms of alantolactone *in vivo*.

Cancers	Animals	Modulated factors	Biological effects	References
Colorectal cancer	Six-week-old female Balb/c mice female sprague-dawley rats	HMGB1, CRT, MHCII, CD86, macrophages, MDSCs, TNF-α, IFN-γ	Promoting antitumor response, suppressing cell proliferation, inducing apoptosis	Zhang et al. (2019a)
	Five-week-old female athymic BALB/c mice	JNK, p38, MAPK, Ki-67		Cao et al. (2019)
Breast cancer	Chick embryo CAM BALB/c nude mice	VEGFR2phosphorylation, PLCγ1, FAK, Src, Akt	Inducing apoptosis, causing cell cycle arrest suppressing growth of xenograft tumors	Liu et al. (2018b)
	MDA-MB-231 xenografts in nude mice	Bcl-2, Bax, caspase-3, cyclinB1, Cdc2, ATF4, CHOP, ki-67		Yin et al. (2019)
	Female athymic BALB/c nude mice	STAT3, MAPKs, NF-κB, IL-6, EGFR, cyclin D1, c-Rel, p65, p50, JNK/AP-1		Chun et al. (2015)
Leukemia	BV173 xenograft nude mouse model	AP2M1, Beclin1, LC3-II/LC3-1, p62, Bax, cleaved caspase 3, cytochrome C, Bcl-2	Inhibiting cell proliferation, inducing apoptosis, inducing cell cycle arrest	Shi et al. (2020)
	B-ALL mice model (NOD-SCID mice)	PARP-1, capase-3, caspase-8, caspase-9, NF-κB, BCR-ABL, EGFR		Xu et al. (2019b)
Pancreatic cancer	Female nude BALB/c mice	TFEB, CTSB/CTSD	Inducing apoptosis, improving chemosensitivity	He et al. (2018)
	Female Wild-type BALB/c mice	STAT3		Zheng et al. (2019)
Gastric cancer	Athymic BALB/c nu/nu female mice	TrxR1, p38MAPK, p38, Ki-67, Bcl-2	Inhibiting proliferation, inducing apoptosis	He et al. (2019a)
Glioblastoma	BALB/c nu/nu male nude mice	IKKβ/NF-κB, p50, p65, p300, COX-2, cytochrome c, cyclin D1, CDK4, MMP-2, MMP-9, caspase-3/9, PARP, Bax, Bcl-2	Inhibiting cell growth, inducing apoptosis	Khan et al. (2012), Wang et al. (2017)

## 2 The Action of ALT Against Human Cancers

### 2.1 Lung Cancer

Lung cancer is one of the most frequent human malignancies worldwide, causing about 1.6 million deaths annually. Risk factors of lung cancer include second-hand smoking, air pollution, genetic reason, etc. (Wu et al., 2020; Yang et al., 2020). In addition, non-small cell lung cancer, accounting for ∼85% of lung cancer cases, is increasing in both incidence and mortality. Non-small cell lung cancer is divided into two histological subtypes, namely lung adenocarcinoma and lung squamous cell carcinoma (Chen et al., 2020; Tubio-Perez et al., 2020). Nowadays, the potential therapeutic effects of traditional medicine, like ALT on patients with both subtypes of non-small cell lung cancer have been studied. It has been found that ALT effectively induces cell apoptosis in both lung squamous carcinoma cells (SK-MES-1) and lung adenocarcinoma cells (NCI-H1299 and Anip973) and the cytotoxic influence of ALT is closely related to the improved treatment efficacy and prognosis of patients with lung cancer (Zhao et al., 2015; Liu et al., 2019). It has also been found that ALT could significantly enhance the anticancer effects of chemotherapy drug gemcitabine on lung adenocarcinoma cells A549 and lung squamous carcinoma cells NCI-H520 cells through inhibiting the activation of AKT/glycogen synthase kinase (GSK) 3β and endoplasmic reticulum (ER) stress pathways (Wang J. et al., 2019). After treatment on A549 lung adenocarcinoma cells, ALT performs the biological functions to trigger oxidative stress mediated-cell apoptosis by abrogating the glutathionylation-dependent STAT3 activation (Maryam et al., 2017). The above studies show the molecular mechanism and biological significance of ALT in the treatment of lung cancer.

### 2.2 Liver Cancer

Liver cancer, with a high death rate and poor 5-years survival, is considered to be one of the most malignant cancers in the world (Feng et al., 2020). The factors leading to liver cancer are as follows: infection of hepatitis B virus (HBV), infection of hepatitis C virus (HCV), alcohol abuse, and alternations of genetic and epigenetic events (Zhang et al., 2020b). There are many strategies to treat liver cancer, such as chemotherapy, radiotherapy, molecular targeted therapy, surgical resection, and liver transplantation (Petrowsky et al., 2020). However, the prognosis is unsatisfactory because of the complex risks and pathological factors (Zhang et al., 2020a; Ruan et al., 2020). Therefore, a new treatment is needed. A recent study has explored the mechanism of ALT-mediated apoptosis in liver cancer cells HepG2 and found that through down-regulating reactive oxygen species (ROS)-mediated alpha serine/threonine-protein kinase (AKT) activation and weakening PTEN induced putative kinase 1 (PINK1)-mediated cell mitophagy, ALT treatment could induce apoptosis in HepG2 cells (Kang et al., 2019). It has also been shown that mitochondrial membrane in HepG2 cells loses the potential when being exposed to ALT and ALT induces apoptosis through modulating the levels of several apoptosis-associated proteins, including Bax, Bak, caspases, etc. (Lei et al., 2012). Another study has drawn a similar conclusion that ALT treatment could enhance Bax/Bcl-2 ratio, promote caspase-3 activation and elevate ROS generation, contributing to inducing apoptosis of HepG2 cells. The abnormally over-expressed and activated signal transducer and activator of transcription 3 (STAT3) signaling pathway have also been proved to be impaired by ALT in liver cancer cells (Khan et al., 2013). These studies indicate that ALT has the potential to be a leading chemotherapeutic candidate in the treatment of liver cancer.

### 2.3 Colorectal Cancer

At present, colorectal cancer ranks as the fourth most deadly cancer in the world. The incidence and mortality of colorectal cancer are much higher in developing countries than in developed countries because of the differences in medical service quality (Suliman et al., 2019; Almatroudi, 2020). It has been found that the incidence of colorectal cancer has a younger trend (The Lancet, 2017; The Lancet Gastroenterology, 2018). Colorectal cancer is a heterogeneous disease with many molecular subtypes, which is beneficial to the prognosis and immunotherapy of cancer (Becht et al., 2016; Wirth and Schneider, 2016). Nowadays, many traditional Chinese medicines (TCM) have been applied to the clinical therapy of cancers. Quercetin synergized with ALT could significantly induce immunogenic cell death (ICD) in colorectal cancer cells. This synergistic therapeutic effect is capable of reversing the immune-suppressive tumor microenvironment, thereby improving cell toxicity and antitumor immunity (Zhang J. et al., 2019). Ding et al. have explored the underlying molecular mechanism of ALT in human colorectal cancer cells SW480 and SW1116 and found that after ALT treatment, the accumulation of ROS causes oxidative DNA damage, contributing to the intrinsic apoptosis pathway of cancer cells (Ding et al., 2016). In addition to causing oxidative DNA damage, ALT could strengthen the effects of oxaliplatin in HCT116 and RKO cells by inducing the activation of MAPK-JNK/c-Jun pathway, deactivation of the JNK pathway, inhibition of p38 MAPK pathway and decrease of intracellular ROS, as has been suggested by two independent studies. The two studies suggest that ALT could suppress cell proliferation and exhibit anticancer effects on colorectal cancer HCT-8 cells and HCT-116 cells (Shi et al., 2011; Babaei et al., 2021; Ren et al., 2021). Besides, ALT could exert the dose-dependently cytotoxic effects on RKO human colon cancer cells and induce cell apoptosis through modulating ROS-mediated mitochondria-dependent pathway (Zhang et al., 2013). The above studies show that ALT treatment could be clinically applied for patients with colorectal cancer in the future.

### 2.4 Breast Cancer

Breast cancer is a common cancer in women (Liu Y. et al., 2020; Wan et al., 2020). Although the diagnosis strategies like the mammogram, have been developed in recent years, the mortality rate of breast cancer is still high (Ranjkesh et al., 2020; Xu et al., 2020). As a result, innovative alternatives are needed to improve the therapeutic outcome of patients with breast cancer Studies have shown that ALT changes the cell morphology and decreases the cell viability of MDA-MB-231 and MCF-7 breast cancer cells (Liu J. et al., 2018; Cui et al., 2018). Administration of ALT can promote apoptosis and suppress migration of MCF-7 cells, which may be due to the decrease of p38 MAPK, NF-κB and nuclear factor E2-related factor 2 (Nrf2) signaling pathways (Liu J. et al., 2018). Liu et al. have revealed that ALT treatment is effective in inhibiting the motility, migration, and tube formation of human umbilical vein endothelial cells (HUVEC), which promote tumor angiogenesis. Besides, ALT impairs the angiogenesis and tumor growth by down-regulating vascular endothelial growth factor receptor 2 (VEGFR2) phosphorylation level and its downstream protein kinases, including phospholipase C gamma 1 (PLCγ1), protein tyrosine kinase 2 (FAK), SRC, and AKT (Liu Y. R. et al., 2018). Triple-negative breast cancer is one of the most challenging subtypes of breast cancers with a high probability of relapse, distant metastasis, and poor survival (Kim et al., 2018; Garrido-Castro et al., 2019). Therefore, analyzing the correlation of ALT and the anti-tumor potential in TNBC is potentially important. Yin et al. have shown that ALT promotes cell death and inhibits cell proliferation of triple-negative breast cancer cells by inducing ROS generation and subsequent ROS-dependent ER stress. Further analyses have shown that thioredoxin reductase 1 (TrxR1) expression and activity are weakened by ALT (Yin et al., 2019). Furthermore, other studies have demonstrated that ALT, serving as a STAT3 inhibitor, suppresses cell migration and the growth of triple-negative breast cancer cells both *in vitro* and *in vivo* (Chun et al., 2015; Kim et al., 2017), highlighting the therapeutic potential in breast cancer treatment.

### 2.5 Leukemia

Leukemia is a malignant progressive disease characterized by abnormal proliferation of haemopoietic stem cells (Abdellateif et al., 2020) and can be divided into four subtypes, namely acute myeloid leukemia, acute lymphoblastic leukemia, chronical myeloid leukemia, and chronical lymphoblastic leukemia. Chronical lymphoblastic leukemia is the most common one that occurs in adults (Hallek et al., 2018; Bosch and Dalla-Favera, 2019), whereas acute lymphoblastic leukemia is most commonly observed in children (Nordlund and Syvanen, 2018). Recently, the biological activities of ALT against THP-1 leukemia cells have been investigated and the results show that ALT plays an important role in inhibiting cell viability and inducing mitochondrial apoptosis in THP-1 cells by provoking ROS production and interfering in STAT3, survivin, c-Jun, and p38 MAPK signaling pathways (Ahmad et al., 2021). Shi et al. have also demonstrated that ALT could promote the expression level of adaptor-related protein complex 2 subunit mu 1 (AP2M1) and inhibit cell proliferation, colony formation, and autophagy of acute lymphoblastic leukemia cells in a dose-dependent manner through up-regulating AP2M1 signaling (Shi et al., 2020). Moreover, the n-hexane fraction extracted from *Inula racemosa Hook. f.*, a mixture of active ingredients mainly consisted of ALT, displays an inhibitory effect on leukemia HL-60 cells through enhancing the intrinsic and extrinsic apoptosis pathways without side effects to normal cells (Pal et al., 2010). ALT also induces cytotoxicity on B cell acute lymphoblastic leukemia *in vivo* and *in vitro* by prompting ROS overload and subsequently resulting in ROS-mediated DNA damage (Xu X. et al., 2019). After the evaluation about the potential activity of ALT in imatinib-sensitive and -resistant cells, Wei et al. have concluded that ALT treatment contributes to significant cell apoptosis in both imatinib-sensitive and -resistant leukemia cells, as indicated by the increase of caspases activation and poly (ADP-ribose) polymerase-1 (PARP-1) cleavage (Wei et al., 2013). These studies strongly support the application of ALT in leukemia treatment.

### 2.6 Pancreatic Cancer

Pancreatic cancer is the second leading cause of cancer death in Western countries, especially in the United States (Neoptolemos et al., 2018; Collisson et al., 2019). The treatment of pancreatic cancer is not easy as early diagnosis is hard (Moore and Donahue, 2019) and there are few effective clinical treatment approaches (Halbrook and Lyssiotis, 2017). It has been revealed that the bioactive mixture of ALT and the analogues (allo-ALT and iso-ALT) could exert significant anti-proliferation and anti-migration effects on PANC-1 and SW1990 pancreatic cancer cells (Yan et al., 2020). It has also been shown that the combination of ALT and other treatments could exert synergized cytotoxic effects on pancreatic cancer. For example, when combined with the chemotherapy drug oxaliplatin, ALT might play a crucial role in deducing tumor-killing effects on pancreatic cancer cells through blocking cathepsin B/cathepsin D activation (He et al., 2018). Similarly, Wang et al. have revealed that ALT triggers synergistic lethality with simultaneous PARP-1 inhibition in homologous recombination-proficient cancer cells (Wang et al., 2020), and promotes the therapeutic sensitivity of pancreatic cancer cells to the anti-cancer drugs, including oxaliplatin (He et al., 2018), PARP inhibitor (olaparib) (Wang et al., 2020), epidermal growth factor receptor (EGFR) inhibitors (erlotinib and afatinib) (Zheng et al., 2019), and so on. Therefore, the combination of natural compound ALT and specific anti-cancer agents is a safe and effective strategy for pancreatic cancer treatment.

### 2.7 Other Tumors

Many studies have suggested that ALT could also exhibit cytotoxic effects on other types of cancers. It has been shown that ALT induces apoptosis and triggers cell-cycle arrest in gastric cancer cells through ROS generation and modulation of several ROS-dependent kinase signaling pathways, such as AKT, p38 MAPK, and NF-κB (He W. et al., 2019; He Y. et al., 2019; Zhang and Zhang, 2019). Furthermore, combined treatment of ALT and ferroptosis inducer erastin could exert a synergistic effect on inducing the death of gastric cancer cells (He W. et al., 2019). It has also been demonstrated that ALT exerts concentration-dependent effects on inhibiting proliferation and inducing apoptosis of cervical cancer cells through regulating the Bcl-2/Bax radio, NF-κB pathway, and thioredoxin reductase (TrxR) activation (Zhang J. et al., 2016; Jiang et al., 2016; Zhang Y. et al., 2019). Furthermore, a newly study have reported that ALT could inhibit the progression of HeLa cells via suppressing the expression of BMI1(Sun et al., 2021). Through down-regulating the NF-κB/COX-2-mediated signaling cascades or triggering the cofilin/G-actin signaling, ALT inhibits the growth and induces apoptosis of glioblastoma cells both *in vivo* and *in vitro* (Khan et al., 2012; Wang et al., 2017; Wang X. et al., 2021). The similar tumor-inhibition effects of ALT, accompanied by apoptosis promotion and growth depression, could also be observed in osteosarcoma (Zhang Y. et al., 2020), esophageal cancer (Wang Z. et al., 2021), multiple myeloma (Yao et al., 2015), etc. The above studies explore the underlying molecular mechanism of the biological activity of ALT, contributing to the application of ALT as a promising chemotherapeutic candidate for different kinds of cancers.

## 3 Clinical Perspective of ALT

As an important sesquiterpenoid extracted from a frequently utilized traditional herbal medicine, ALT has been confirmed to possess a broad spectrum of pharmacological properties, including anti-tumor, anti-fungal, and anti-inflammatory activities. Up to now, many studies have reported the anticancer effects of ALT *in vitro* and *in vivo*. However, the biological actions of ALT are easily influenced by some factors, like bioavailability.

Recently, a pharmacokinetics study has suggested that the oral bioavailability of ALT is quite low, which is one challenge in clinical trial design to explore the biological actions. Some defects of ALT, such as low water solubility, limit the absorption and bioavailability *in vivo* (Xu et al., 2015). Low oral bioavailability probably results from intestinal metabolism, poor permeability, and low aqueous solubility (Zhou et al., 2018). However, according to the compatibility principle in the Prescription Dictionary of Chinese Medicine, the combination of ALT and other herbs could effectively reduce the toxicity and enhance intestinal absorption, contributing to stronger bioavailability and therapeutic actions (Xu R. et al., 2019). It is well known that evaluation of intestinal bacteria is one challenge in clarifying the metabolism of oral drugs (Zimmermann et al., 2019). A biotransformation strategy based on the anaerobic culture of intestinal bacteria has been developed by Yao et al. for identifying ALT metabolites (Yao et al., 2016). In addition, ALT-entrapped nanostructured carriers have been developed to improve the bioavailability and potential cytotoxicity efficacy of ALT against cancers (Zhang J. et al., 2019). These studies are beneficial for the evaluations of ALT application in the future. Unfortunately, until now, there are no clinical trials to explore the bioavailability and anti-tumor effect of ALT in cancer patients. Therefore, to verify the pharmacological activities of ALT, more investigations, especially well-designed clinical trials, remain to be determined in the future.

## 4 Implication of ALT for Cancer-Associated Signaling Pathways

As shown in previous studies, ALT has good clinical prospects as therapeutic agents for human cancers. It has been found that ALT exerts high cytotoxicity effects, such as anti-proliferation, anti-metastasis, and pro-apoptotic cascades on many human cancer cell lines through interfering with several molecular events (Zhang J. P. et al., 2016; Nadda et al., 2020).

Previous studies have illustrated the important roles of ROS in maintaining the stable microenvironment of tissues and affecting the genesis and development of malignant tumors (Ippolito et al., 2020; Shen et al., 2020). If the ROS production is not in balance, the extensive damage response in cells caused by oxidative stress would result in higher risks of diseases, like diabetes, cardiovascular disease, cancers, etc. (Tavares and Seca, 2019). Therefore, keeping the balance of ROS levels is beneficial for regulating cancer treatment efficacy (Jiang et al., 2019; Zhou et al., 2020). It has been found that ALT could increase the concentration of ROS and trigger the intrinsic apoptosis pathway of colorectal cancer cells (Ding et al., 2016). Kang et al. have reported that ALT could induce cell-cycle arrest and cell apoptosis in HepG2 cells by regulating intracellular ROS accumulation, which provides a new strategy to treat liver cancer (Kang et al., 2019).

In addition, as a transcription factor, NF-κB is related to the regulation of carcinogens, such as promoting cell proliferation, regulating apoptosis, facilitating angiogenesis, and stimulating metastasis (Liu Z. et al., 2020; Espinosa-Sanchez et al., 2020). NF-κB also modulates the immune and inflammatory responses, influencing cancer cell growth (Fusella et al., 2017; Taniguchi and Karin, 2018). Effective regulation of the activation of the NF-κB signaling pathway is significant in developing chemotherapies. It has been found that ALT-targeted NF-κB and the downstream signaling pathways inhibit the migration of breast cancer cells and trigger the apoptosis of chronical myeloid leukemia cells (Wei et al., 2013; Liu J. et al., 2018). It has also been demonstrated that ALT promotes cell apoptosis in acute lymphoblastic leukemia and gastric cancer through inhibiting NF-κB activation (He Y. et al., 2019; Xu X. et al., 2019). Besides, ALT significantly delays the cell proliferation of HeLa cells in a dose-dependent manner through targeting NF-kB signaling pathways (Zhang Y. et al., 2019).

It is well-known that clarifying the underlying functions of VEGFR contributes to the understanding of the angiogenesis and therapeutic response of cancer cells (Haibe et al., 2020; Kratzsch et al., 2020). Furthermore, VEGF plays a crucial role in the development of molecular-targeted treatment or other novel anti-cancer drugs in clinical practice (Apte et al., 2019). Liu et al. have uncovered that ALT inhibits VEGFR2 phosphorylation, and impairs VEGF-VEGFR2 signaling in HUVECs (Liu Y. R. et al., 2018). ALT could also reduce VEGF secretion, thereby suppressing the adhesion of multiple myeloma cells (Yao et al., 2015). These findings suggest that ALT may be a promising agent to fight against angiogenesis and invasion in cancers through intervening in VEGF-VEGFR pathways.

The aberrant activation of the p38 MAPK signaling pathway is involved in various biological processes, facilitating the development and treatment of cancer (Wang K. et al., 2019; Reger de Moura et al., 2020). As an essential regulating factor, p38 MAPK participates in many cellular activities, making cancer cells perceive and adapt to environmental stress signals (Low and Zhang, 2016; Martinez-Limon et al., 2020). Studies have shown that deactivating the p38 MAPK pathway could facilitate the ALT-mediated cell apoptosis in colon cancer cells and breast cancer cells (Liu J. et al., 2018; Cao et al., 2019). Moreover, ALT exerts attractive pharmacological activities on lung cancer cells by blocking the p38 MAPK pathway (He W. et al., 2019; Liu et al., 2019). He et al. have further revealed that ALT modulates the ROS-mediated p38 MAPK pathway and induces cell apoptosis in gastric cancer. More importantly, ALT treatment markedly enhances the cell sensitivity to the ferroptosis inducer erastin (He W. et al., 2019).

In addition, there are a few studies concerning about the correlation between ALT administration and cell autophagy in cancer cells. ALT could play a significant role in promoting impaired autophagy, facilitating to allay osteoarthritis and strengthen pancreatic cancer cells’ chemosensitivity (He et al., 2018; Pei et al., 2021). Another two studies have demonstrated that treatment with ALT could significantly downregulate the cell autophagy in ALL and liver cancer cells, implying that ALT have the potential to kill cancer cells through modulating autophagy (Kang et al., 2019; Shi et al., 2020).

Taken together, accumulating reports have showed that ALT exerts anticancer effects on various kinds of cancers, such as liver cancer, colorectal cancer, breast cancer, etc. And the potential molecular mechanisms involved in ALT’s anticancer activities are inhibiting JNK and p38 MAPK pathways, PI3K/AKT/GSK3β pathways, NF-κB/COX-2 pathways and promoting cell apoptosis-associated signalings. These findings above-mentioned demonstrate that ALT may be a potent therapeutic candidate for cancer reseach and treatment. However, more comprehensive studies are still needed to further explore the detailed functions of ALT.

## 5 Conclusion

In summary, the exploration of agents from plants will help to develop new therapeutic strategies and drugs in future clinical treatment. ALT possesses superior anti-tumor properties besides anti-inflammatory and antimicrobial activities and can be a potential drug candidate for cancer therapy. From some experiments of ALT *in vivo* and *in vitro*, we can know that ALT can synergize with chemical drugs to enhance their anticancer effects, such as Quercetin and oxaliplatin. Additionally, it was reported that ALT could enhanced the therapeutic sensitivity on cancer treatment. Although there are some studies concerning the cytotoxic effects of ALT *in vivo* and *in vitro*, more profound investigations are still needed to clarify the underlying mechanisms of ALT in the treatment of human malignancies. Besides, accurate and reliable clinical research, for example, randomized controlled trials, are needed to prove the effectiveness of ALT as a therapeutic agent for cancers.

## Abbreviations

ALT, alantolactone; AKT, alpha serine/threonine-protein kinase; AP2M1, adaptor-related protein complex 2 subunit mu 1; EGFR, epidermal growth factor receptor; GSK, glycogen synthase kinase; HBV, hepatitis B virus; HCV, hepatitis C virus; HUVEC, human umbilical vein endothelial cells; ICD, immunogenic cell death; MAPK, Mitogen-activated protein kinases; Nrf2, nuclear factor E2-related factor 2; PINK1, putative kinase 1; PLCγ1, phospholipase C gamma 1; ROS, reactive oxygen species; STAT3, signal transducer and activator of transcription 3; TCM, traditional Chinese medicines; TrxR1, thioredoxin reductase 1; VEGFR2, vascular endothelial growth factor receptor 2.
